# From methods to policy: a critical appraisal of the 2024 ECDC guidance on enhanced influenza vaccines

**DOI:** 10.3389/fpubh.2026.1760791

**Published:** 2026-03-11

**Authors:** Raúl Ortiz de Lejarazu, Daniel Ocaña, Pilar Arrazola, Alejandro Orrico-Sánchez, Esther Redondo, Iván Sanz-Muñoz, Natividad Tolosa, Antoni Trilla

**Affiliations:** 1 National Influenza Center, Valladolid, Spain; 2 Primary Care Center Algeciras-Norte, Algeciras, Spain; 3 Department of Preventive Medicine, Hospital Universitario 12 de Octubre, Madrid, Spain; 4 Vaccine Research Area, Foundation for the Promotion of Health and Biomedical Research of the Valencian Community (FISABIO), Valencia, Spain; 5 Internacional Health Center Madrid Salud, Madrid, Spain; 6 Preventive Medicine, Hospital Universitari i Politècnic La Fe, Valencia, Spain; 7 Hospital Clinic de Barcelona, Barcelona, Spain; 8 University of Barcelona, Barcelona, Spain; 9 Barcelona Institute for Global Health (ISGlobal), Barcelona, Spain

**Keywords:** ECDC, inclusion criteria, influenza, methodological consistency, review, vaccine

## Introduction

The recent European Centre for Disease Prevention and Control (ECDC) review of influenza vaccines, published in 2024 (ECDC-2024) (1) provides an important opportunity to reflect on how methodological choices in evidence synthesis can shape health technology assessment outcomes in the European Union. Because vaccine evaluations increasingly inform regulatory, reimbursement, and policy decisions under the European Union Regulation on Health Technology Assessment (EU HTAR) framework, inconsistencies in study appraisal and analytical approaches may have implications that extend beyond an intervention. Using the ECDC-2024 influenza vaccine review as a case study, this editorial highlights key methodological concerns and discusses why rigor and transparency are essential for vaccine assessments across the EU.

Influenza is a major global public health threat, and vaccination is the most effective preventive measure. Each year, approximately one billion seasonal influenza cases are reported, including between three and five million severe cases and between 290,000 and 650,000 deaths (2). In Spain, influenza causes an average of 29,000 hospitalizations, 2,200 intensive care unit admissions, and around 1,600 deaths per season, with a hospitalization rate of 62.1 per 100,000 inhabitants. This rate is similar to that reported in Norway and the United Kingdom, but higher than that reported in France and Portugal (3).

Unlike most medical interventions, vaccines are used for prevention and are administered to healthy individuals, including children and people who are frail or have comorbidities. Consequently, vaccines are subject to a more complex decision-making process and are commonly included in a country's National Immunization Program (NIP) before being administered to the target population(s).

The EU HTAR was adopted in December 2021 to consolidate and harmonize processes and clinical assessments across all EU member states (4). The EU HTAR will be implemented gradually, starting with oncology medicines and advanced therapy medicinal products in January 2025, and extending to orphan drugs from January 2028. From January 2030 onwards, all other centrally approved medicines in the EU, including vaccines, will also fall under the scope of this legislation. Meanwhile, the evaluation of vaccines and the implementation of immunization programs remain the responsibility of National Immunization Technical Advisory Groups (NITAGs). In Spain, this role is carried out by the *Ponencia de Programa y Registro de Vacunaciones* (5), a specialized body composed of experts who provide evidence-based recommendations on the inclusion of vaccines in the NIP.

Although the ECDC does not have the mandate to issue legally binding recommendations, it plays an important role in advising member states. In fact, the decisions issued by NITAGs in different countries partially rely on clinical evidence synthesized in literature reviews such as those produced by the ECDC. Spanish NITAG, for example, updates vaccination recommendations based on, among other sources, technical documents and systematic literature reviews (SLRs) published by the ECDC (6).

Given the importance of ECDC SLRs in informing recommendations across EU member states, it is essential that they maintain methodological robustness throughout their development. A thorough assessment should be undertaken to identify the main sources of heterogeneity across studies and inform the selection of the most appropriate methods for data analysis. A lack of clarity and consistency could hamper its practical application, especially if used to inform decision-making.

An example of how methodological heterogeneity can limit the scope of analyses is the recent update of the ECDC SLR on the safety, efficacy, and effectiveness of influenza vaccines (1). In 2020, the ECDC completed an SLR (ECDC-2020) on the efficacy, effectiveness, and safety of influenza vaccines in adults (≥18 years) (7). Its update, Askar et al. (1) (ECDC-2024), was highly anticipated by influenza experts from NITAGs and clinical professionals, as it provides a concise scientific synthesis to support national policies.

NITAGs noted the methodological improvements introduced in ECDC-2024. The update narrowed outcomes to laboratory-confirmed clinical criteria and prioritized randomized trials. Additionally, the use of the ROBINS-I tool to assess bias in non-randomized studies improved quality assessment. Finally, the input from experts from several EU and European Economic Area (EEA) member states contributed to the evaluation process.

Although the report has many strengths, independent evaluations by several working groups, including ours, have identified some concerns that warrant attention. Given their potential impact on decision-making, these concerns could help optimize the influenza vaccination recommendations. The aim of this article is therefore to highlight several aspects of the ECDC-2024 review that might have warranted a more detailed analysis and a broader consensus among stakeholders. Other expert groups (8) have also identified limitations that deserve attention due to their potential impact on decision-making.

## Nuances in GRADE assessments

A key concern lies in the evaluation that assigned Grading of Recommendations Assessment, Development, and Evaluation (GRADE) certainty levels to the included studies.

The methodology applied in the review, based on the GRADE framework, inevitably involves a degree of subjectivity, as certainty ratings depend in part on the evaluators' judgment. While indirect treatment comparisons (ITCs) and observational studies are gaining importance in vaccine evaluation due to the shortage of direct comparisons, double-blinded randomized controlled trials (RCTs) remain the gold standard for defining clinically meaningful differences, including for vaccines. However, this growing reliance on observational evidence does not, in our view, justify assigning a “moderate” level of evidence in the GRADE system for the observational study by Domnich et al. (9) in ECDC-2024. Even if this rating may be debatable, it would have been beneficial to provide a discussion of the study's methodological limitations and potential sources of bias. This study includes a sample size of *n* = 512 (83 cases and 429 controls) and applies propensity score matching to adjust for covariates. However, supplementary table S1 of the aforementioned reference shows that several covariates (age, season, diabetes, among others) retain standardized mean differences (SMD) greater than 0.10 after matching, indicating insufficient covariate balance between groups. This cutoff has been widely recommended by Austin (10) and Stuart (11) as a minimum criterion for adequate adjustment; therefore, SMD values above 0.10 suggest the presence of potential residual confounding bias.

This issue becomes more relevant when the evidence level of an RCT, such as the one by Díaz-Granados et al. (12), was downgraded mainly due to its industry funding. Despite the importance of transparency in research funding, large pivotal trials remain predominantly industry-funded, with close to 90% relying on pharmaceutical sponsorship (13–15). Díaz-Granados et al. conducted a large, multicenter, double-blind, randomized controlled trial comparing high-dose vs. standard-dose influenza vaccine in older adults. The randomized allocation and double-blinding procedures reduce selection, performance, and detection bias, while the active comparator design ensures clinical relevance. The large sample size (31,989 participants enrolled from 126 research centers) and prespecified efficacy endpoints enhance statistical power and internal validity, supporting the robustness of the trial's causal inference regarding vaccine efficacy. Re-examining whether sponsorship alone justifies a lower rating could help ensure that robust data receive the proportional weight they deserve.

## Inclusion criteria and reporting consistency

A further analysis identified that several trials included in ECDC-2024 (16–18) did not fully align with the established eligibility criteria.

In some cases, the reported figures differed from those published in the original sources. Thus, for Van Buynder et al. (19), Table 21 reports rVE 42% (95% CI −8 to 69), whereas the original article reports rVE 63% (95% CI 4–86) derived from aOR = 0.37 (95% CI 0.14–0.96). Mira-Iglesias et al. study reports rVE 19% (95% CI −10 to 41%) in Table 21, but calculation from the original data yields 2.3%. Pebody et al. (16) provides rVE 16% (95% CI −176 to 75%) for the 2018–2019 season, though only absolute VE is reported in the publication. Bellino (21) shows rVE −1% (95% CI −122 to 59%) in Table 21, corresponding to adjusted IVE for adults ≥65 in the original article. The results for Rondy (27) are not found in the publication. Finally, the reference Rondy (18) appears incorrect (likely Kissling 2017) and the reported rVE in Table 21 is neither reported nor calculable from the study data.

In others, the criteria for highlighting or overlooking certain outcomes were not always explicit (17–21). This is the case of one study that appears to have been excluded for lacking laboratory confirmation, despite the article providing such verification (22). We believe that clarifying the inclusion criteria could improve transparency.

## The importance of the PICO Question

A key element in evidence synthesis is the formulation of the PICO (Population, Intervention, Comparator, Outcomes) question, which sets the framework for the literature search. Compared with ECDC-2020, the PICO framework used in ECDC-2024 was more restrictive, tightening both inclusion and exclusion criteria. Two major changes are particularly noteworthy. First, the exclusion of non-influenza vaccine comparators and second, the restriction to studies reporting only laboratory-confirmed outcomes.

By limiting comparators to other influenza vaccines, the review excluded several robust RCTs with laboratory-confirmed outcomes for adjuvanted formulations, particularly the only individual RCT of an adjuvanted vaccine (23) and the DANFLU-1 trial (24), which demonstrated that the high-dose vaccine reduces influenza-related hospitalizations.

Furthermore, by narrowing the PICO question to laboratory-confirmed influenza alone, ECDC-2024 omitted clinically relevant outcomes, such as hospitalization, complications, and post-discharge mortality, which better gauge the disease burden. While prioritizing laboratory confirmation strengthens diagnostic specificity, excluding these broader outcomes could underestimate the overall contribution of vaccines in preventing severe influenza-related complications.

## Conclusions

The introduction of a new revised legislative framework, such as the EU HTAR, which aims to harmonize vaccination decisions, may provide an opportunity to standardize vaccine assessment methods across the EU, including the development of best-practice recommendations or a vaccine-specific methodological framework for conducting and interpreting systematic reviews.

ECDC-2024 (1) demonstrated certain methodological shortages that support the need for a better tracking system of the methodology used, especially considering that its conclusions had already guided the decision-making process by some public NITAGs (25, 26).

Our group of experts considers it essential to highlight these issues to avoid an uncritical transposition of EU recommendations into national policies. Therefore, we advocate for the adoption of more robust and harmonized working methodologies to ensure that influenza vaccination strategies are firmly based on sound clinical evidence.
